# Aflatoxin B1 Acts as an Effective Energy Donor to Enhance Fluorescence of Yellow Emissive Carbon Dots

**DOI:** 10.1021/acsomega.2c03498

**Published:** 2022-08-08

**Authors:** Özge Ergüder, Sultan Şahin Keskin, Ilgın Nar, Levent Trabzon, Caner Ünlü

**Affiliations:** † Department of Nanoscience and Nanoengineering, Istanbul Technical University, Maslak, 34469 Istanbul, Turkey; ‡ Istanbul Technical University Nanotechnology Research and Application Center (ITUNano), 34469 Istanbul, Turkey; § MEMS Research Center, Istanbul Technical University, 34469 Istanbul, Turkey; ∥ Faculty of Mechanical Engineering, Istanbul Technical University, 34469 Istanbul, Turkey; ⊥ Faculty of Science and Letters, Department of Chemistry, Istanbul Technical University, Maslak, 34469 Istanbul, Turkey

## Abstract

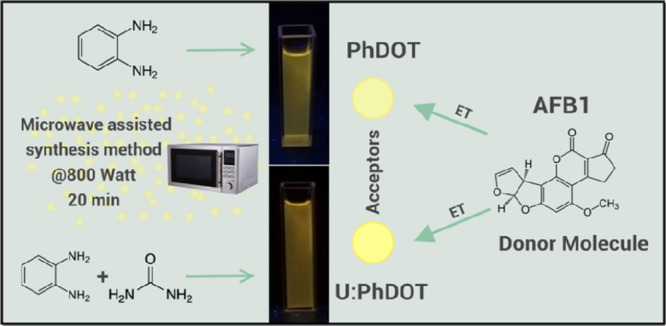

Carbon dots (CDs) are versatile fluorescent nanocrystals with unique optical and structural properties and are commonly used in biosensing, bioimaging, and biomolecule tagging studies. However, fluorescence of CDs is brightest in the wavelength range of 430–530 nm, which overlaps with the autofluorescence range of many eukaryotic cells and makes CDs impractical for in vivo and in vitro imaging studies. Thus, the design of yellow-red emissive CDs with high quantum yield is of importance. In this study, the quantum yield of traditional yellow emissive CDs was enhanced by two different methods: (1) the surface of traditional yellow emissive CDs passivated with a biomolecule, urea, through easy, rapid, inexpensive microwave assisted synthesis methods and (2) a fluorescent biomolecule, aflatoxin B1, used as an energy donor for yellow emissive CDs. In the first method, the quantum yield of the CDs was enhanced to 51%. In the second method, an efficient energy transfer (above 40%) from aflatoxin B1 to the CDs was observed. Our study showed that highly luminescent yellow emissive CDs can be synthesized by simple, rapid microwave assisted synthesis methods, and these CDs are potential candidates to sense aflatoxin B1. Furthermore, our results indicated that Aflatoxin B1 can be considered as an emission booster for CDs.

## Introduction

Carbon dots (CDs) are versatile fluorescent carbon based nanomaterials that exhibit quantum confinement effect properties and possess exclusive properties such as bright fluorescence, low toxicity, cost-effective production, biocompatibility, chemical inertness, etc. With their unique optical properties, CDs show great potential in a variety of applications, especially in sensor development,^1^ bioimaging,^2^ drug delivery,^3−6^ light emitting devices,^7,8^ and photocatalytic technology.^9,10^ In applications regarding biotechnology, CDs are more preferable to quantum dots (QDs) containing heavy metals because of their biocompatibility and environmentally friendly production. However, CDs emit fluorescence in the blue-green color range (430–530 nm) with the highest quantum yield (QY, an indicator of the brightness of a fluorescent material) and as a consequence have a limited applicability in in vivo imaging because this color range overlaps with the autofluorescence of most of the eukaryotic cells (420–520 nm).^11−14^ Therefore, developing yellow-red emitting CDs (emission wavelength above 550 nm) with high QYs is of importance and studied widely.

Generally, 1,2-phenylenediamine (1,2-PD) and its isomers were used as carbon precursors to produce yellow-red emissive CDs. CDs obtained by using PD as the carbon precursor were used in different applications such as cell imaging,^15,16^ sensing H_2_O_2_,^17^ Cu^2+^ ions,^18,19^ Fe^3+^ ions,^15,16^ CrO_4_^2–^,^20^ atrazine,^21^ glutathione,^18^ cystine, and urine,^22^ and production of white emitting diodes.^23^ Bottom up approaches such as the hydrothermal synthesis method and the microwave assisted synthesis method are frequently used to synthesize CDs by using PD and its isomers as carbon precursors. N, S-codoped green emissive CDs (QY = 64.03%) from m-PD was synthesized by the hydrothermal method and used in cell imaging.^24^ Full color fluorescent CDs from m-PD and H_3_PO_4_ were synthesized by the hydrothermal method and dissolved in different solvents.^25^ In one of the last published studies, CDs derived from 1,2-PD by different oxidants and acids via protonation–deprotonation were produced by microwave assisted hydrothermal methods on a large scale.^26^ Also, in a very recent study it was shown that urea can be used to passivate the surface of 1,2-PD derived CDs through hydrothermal synthesis methods and enhance the QY of the quantum dots.^27^

In this study, we passivated the surface of traditional yellow emissive CDs through a facile, rapid, and energy efficient microwave-assisted synthesis method using 1,2-phenylenediamine as the carbon precursor and urea as the passivating agent. The NH_2_ groups in urea passivated the surface of the CDs and caused a significant increase in the QY (from 40% to 51%) of CDs. Also, aflatoxin B1 (AFB1), a biomolecule with fluorescence properties, was used as an energy donor to increase the quantum yield of CDs furthermore. Our results revealed that yellow emissive CDs had great potential in sensing AFB1. Also, we have shown that AFB1 was an efficient energy donor for yellow emissive CDs with energy transfer efficiency around 0.42 and could be used as an emission-booster for yellow emissive CDs.

## Materials and Methods

### Chemicals

Aflatoxin B1 from *Aspergillus flavus* was obtained from Sigma-Aldrich Co., and 1,2-phenylenediamine (*o*-phenylenediamine), urea, and sodium hydroxide were of the highest purity and were purchased from Merck.

### Synthesis of PhDOTs and U:PhDOTs

A traditional microwave-assisted method was applied to synthesize carbon dots from *o*-phenylenediamine.^15^ For the synthesis of PhDOTs, 400 mg of *o*-phenylenediamine (carbon precursor) was dissolved in 20 mL of ultrapure water and added into a 50 mL flat bottom flask. Then, the homogeneous solution was placed into a kitchen-type microwave oven (SAMSUNG/model no. MS23F300EEK) and heated at 800 MW on a rotating plate for 20 min. For the synthesis of U:PhDOTs, 400 mg of *o*-phenylenediamine (carbon precursor) and 100 mg of urea (passivating agent) were dissolved in 20 mL of ultrapure water, and the same procedure as noted previously was followed without any further modification. At the end of the synthesis procedure, a solid product with a yellowish color was obtained. Then, the solid product was dissolved in 10 mL of ultrapure water, and the first big clusters were removed by a special filter paper with a 10 μm pore size and then filtered through a syringe filter with a 0.22 μm pore size. In the last step, the solvent of the purified carbon quantum dot solution was dried and the purified solid product was kept at room temperature in a dark place for further analysis.

### Interaction of the Carbon QDs with the Aflatoxin B1 Molecule

To understand the interaction between carbon dots and AFB1, solutions with different concentrations of AFB1 (10 μg/mL, 8 μg/mL, 4 μg/mL, 2 μg/mL, 1 μg/mL, 0.5 μg/mL, 0.25 μg/mL, 0.125 μg/mL, and 0.62 ng/mL) were prepared, added to a carbon dot solution (the concentration of carbon dots was kept the same in each aliquot), and left for 20 min for the interaction to complete.

### Optical and Structural Characterization of Carbon Dots, AFB1, and Carbon Dot–AFB1 Complexes

Optical characterizations of AFB1, CDs, and CD–AFB1 complexes were studied by fluorescence spectroscopy, UV–vis spectroscopy, and time-resolved fluorescence spectroscopy (TRF). Fourier transform infrared (FTIR) spectroscopy and Raman spectroscopy (RS) were used for the structural characterization of the CDs. The absorption spectra of PhDOT and U:PhDOT carbon dots and AFB1 were recorded by the Scinco Neosys-2000 double-beam ultraviolet–visible (UV–vis) spectrophotometer. The emission spectra of carbon dots and AFB1 were recorded by the Varian Cary Eclipse fluorescence spectrofluorometer using various excitation wavelengths (λ_exc_) between 350–530 nm. To preclude the errors raised from self-absorption, each sample was dissolved in 10 mL of ultrapure water after purification and then diluted until the optical density was under 0.1. The emission color of each sample was examined under 366 nm UV-light irradiaton. The quantum yields of the PhDOTs and U:PhDOTs at 400 nm carbon dots were measured by using coumarine 102 in ethanol as a standard (QY:0.93).^28^ The hotoluminescence excitation (PLE) spectra of PhDOTs and U:PhDOTs were collected at an emission wavelength (λ_ems_) of 570 nm. The emission spectrum of each sample was collected using excitation wavelengths of 350, 400, 450, and 530 nm. The photostabilities of the CDs were checked by recording the change in intensity of the emission at 570 nm upon continuous illumination with 350 nm (100 mW/cm^–2^) in a 3 mL quartz cuvette under constant stirring at 25 °C for 1 h. The emission spectrum of each AFB1–carbon dots mixture was compared with the emission spectrum of AFB1 to observe differences between emission characteristics of AFB1 and emission characteristics of AFB1–carbon dot mixtures (λ_exc_ = 400 nm). The effect of the interaction between AFB1 and the carbon dots was observed by collecting emission/excitation spectra of AFB1–PhDOTs and AFB1–U:PhDOTs mixtures. All of the optical characterizations were performed on aqueous solutions, and all spectroscopic analyses were conducted at room temperature. The fluorescence kinetics of PhDOTs and U:PhDOTs were measured by a PicoQuant MicroTime 100 time-resolved confocal fluorescence microscope. The excitation beam was provided by an 8 mW picosecond diode laser (λ_exc_ = 375 nm) pulsed at a 60 MHz repetition rate to 40× objective lens. For the spectrally resolved data, bandpass filters with a bandwidth of 15 nm were used. To analyze the structures of PhDOT and U:PhDOT CDs, Fourier transform infrared (FTIR) spectroscopy and Raman spectroscopy (RS) were used. To obtain detailed information about the bonding characterization of PhDOT and U:PhDOT quantum dots, carbon dots were characterized by attenuated total reflectance FTIR (ATR-FTIR) spectroscopy and Raman spectroscopy. The FTIR spectra of the purified carbon dots were measured in the range of 800–4000 cm^–1^ by using a PerkinElmer ATR-FTIR spectrophotometer. Hydrodynamic radius distributions of carbon dots were determined through DLS (Zetasizer Nano ZS, Malvern Instruments Ltd., U.K.).

## Results and Discussion

**Figure 1:**
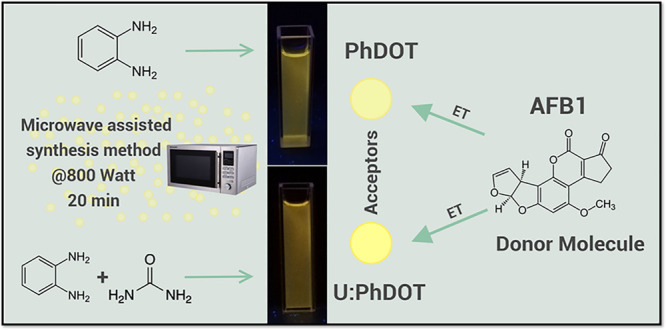
Schematic illustration of the synthesis of carbon dots and their photoluminescence under UV light (366 nm) irradiation.

Most of the carbon dot types fluoresce in the blue-green color range with high brightness, and their brightness decreases in the yellow-red color range. However, the yellow and red emissive fluorescent materials are of interest in biomedical studies because most of the eukaryotic cells have autofluorescence in the range of 350–550 nm, in other words, the blue-green color range. Therefore, development of bright yellow and/or red emissive carbon dots is a hot topic and a challenge. The emission color of carbon dots can be controlled either by controlling the nanoparticle size or by controlling nanoparticle surface characteristics and composition.^29−31^ The size control of carbon dots by using bottom-up synthesis methods can be challenging because of bottom-up synthesis conditions.^29−31^ On the other hand, surface characteristics and composition of carbon dots can be manipulated through bottom-up synthesis relatively easily.^29−35^ Among all types of bottom-up synthesis techniques, the microwave synthesis technique is one of the most preferred synthesis techniques to produce carbon dots with bright fluorescence, in other words, high quantum yield (QY) (Figure 1). Blue-green emissive carbon dots with high quantum yield (above 25%) can be synthesized easily through microwave assisted synthesis techniques; however, there are only a few studies on the synthesis of yellow or red emissive carbon dots by using microwave assisted synthesis techniques.^15,23^ Generally, isomers of phenylenediamine are the most preferred carbon precursors to synthesize yellow-red emissive carbon dots.^22,23,26^ On the other hand, the highest quantum yield for yellow emissive carbon dots synthesized through microwave assisted synthesis methods was observed as 38.5%.^15^

In this study, yellow emissive carbon dots were synthesized according to a well-known method that was described by Song et al.;^15^ 1,2-phenylenediamine was treated at 800 W in a kitchen-type microwave for 20 min, and carbon dots were obtained at the end of treatment. This synthesis method was modified by adding urea to 1,2-phenylenediamine solution before microwave treatment, and as a result, carbon dots with brighter yellow emission were obtained. To uncover the reasons behind the increase the emission brightness of the carbon dots, structural and optical properties of the carbon dots were investigated in detail.

**Figure 2:**
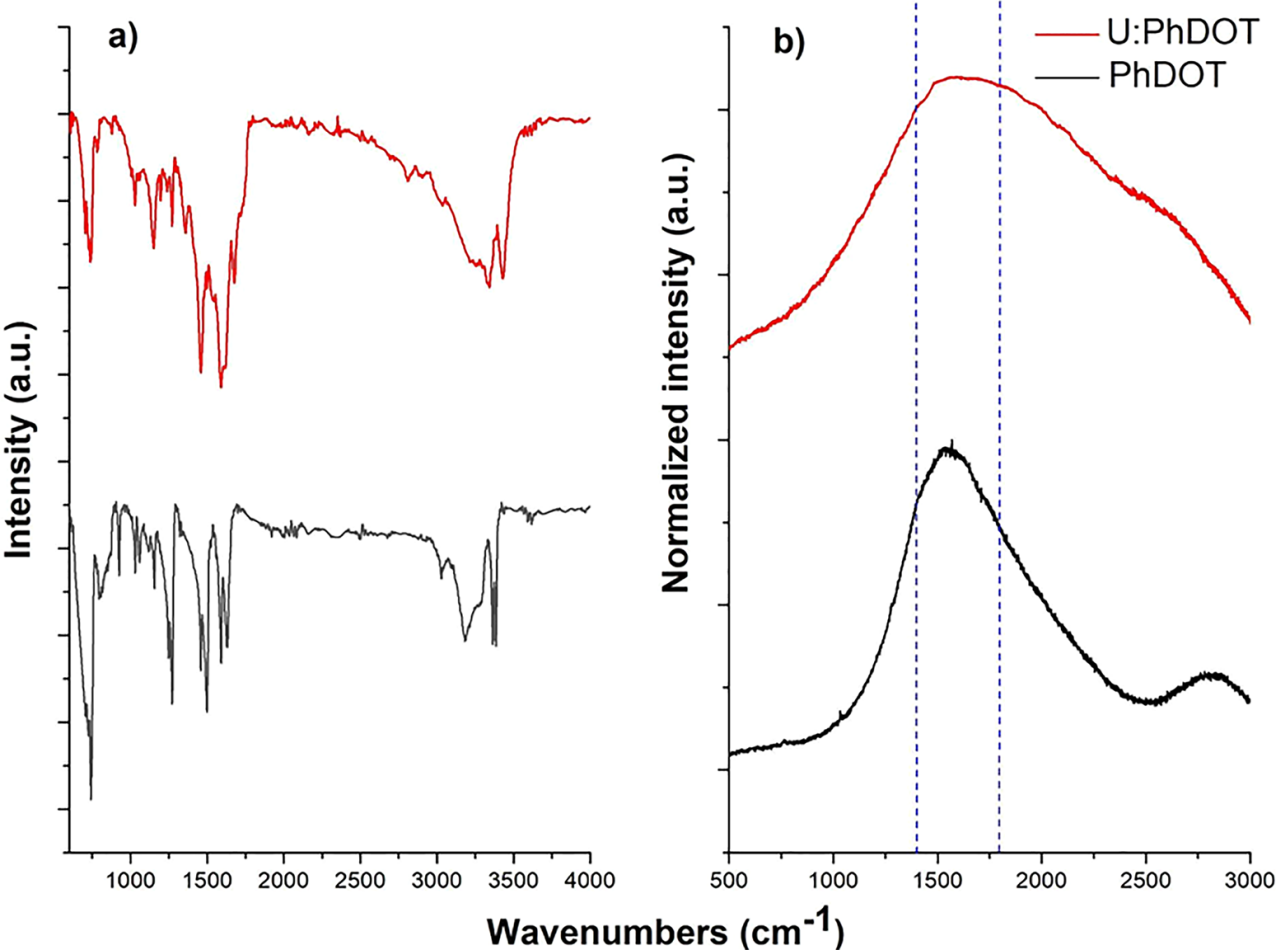
(a) FTIR and (b) Raman spectra of the CDs.

The bonding characterization of the core structure of the carbon dots and the characteristics of functional groups on the surface of the carbon dots were investigated by Fourier transform infrared (FTIR) and Raman spectroscopies. The FTIR spectrum of U:PhDOT and PhDOT displayed characteristic differences (Figure 2). As the major difference, the intense peak at 3450 cm^–1^ in the FTIR spectrum of U:PhDOT corresponding to primary amines on the surface of the carbon dot could not be observed in the FTIR spectrum of PhDOT (Figure 2a). On the other hand, both the FTIR spectrum of U:PhDOTs and the FTIR spectrum of PhDOTs had characteristic peaks corresponding to secondary amines (3300–3400 cm^–1^ band), carboxylic groups (1600–1700 cm^–1^ band), hydroxyl groups (3200–3300 cm^–1^ band), and C–N bonds (1000–1250 cm^–1^) (Figure 2b). The observation of peaks corresponding to C–N bonds and secondary amine groups indicated that both carbon dots possessed nitrogen within the core structure; however, only U:PhDOTs had primary amine group peaks, which showed that only U:PhDOTs had amine groups on the surface. It should be noted that both types of carbon dots possessed FTIR peaks corresponding to carboxylic groups and hydroxyl groups, which indicated the existence of carboxylic groups and hydroxyl groups on the surface. It should be noted that the hydrodynamic radii of PhDOTs and U:PhDOTs were slightly different (12 nm for PhDOTs and 15 nm for U:PhDOTs) (Figure S1). The differences in the hydrodynamic radii of the carbon dots indicated that the surfaces of the PhDOTs and U:PhDOTs had different characteristics. Also, the DLS results of PhDOTs perfectly matched with the TEM results, which were recorded in Song et al.’s study (8–11 nm).^15^

The Raman spectra of Ph:DOT and U:PhDOT also showed drastic differences. The Raman spectrum of each carbon dot possessed a D band around 1400 cm^–1^ and a G band around 1800 cm^–1^, which revealed the graphitic nature of the carbon dots with a considerable amount of surface defects (Figure 2b).^15^ On the other hand, the G:D ratio in the Raman spectrum of U:PhDOT was significantly higher than the G:D ratio in the Raman spectrum of PhDOT (Figure 2b). In the Raman spectrum of graphitic materials, the D band reflects defects on the surface, and the G band rises because of the existence of sp^2^ hybridized carbons.^15^ It should be noted that the defect energy states in carbon dots arise mainly because of the presence of oxygen on the surface, which exists in hydroxyl and carboxylic groups.^36^ In light of this information, it was concluded that PhDOTs had significantly higher amounts of defect states on the surface with an intense D band, which could be due to the dominant presence of carboxylic and hydroxyl groups on the surface, whereas U:PhDOT, which had amine groups on its surface together with carboxylic groups, had fewer surface defects with the presence of an intense G band in its Raman spectrum.

**Figure 3:**
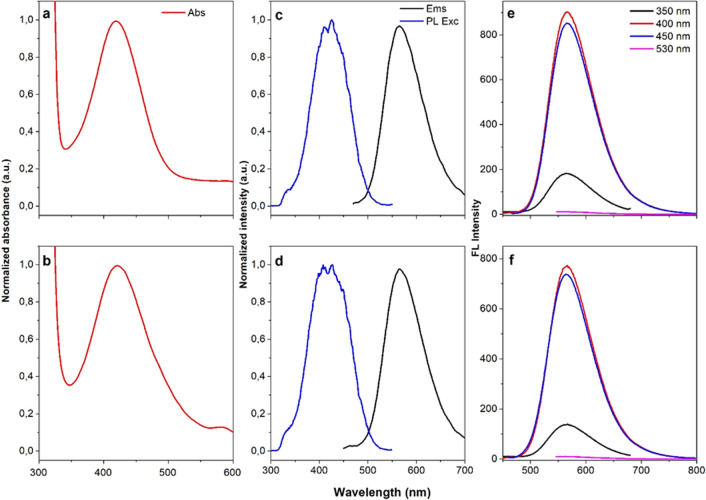
UV–vis spectra of (a) PhDOT and (b) U:PhDOT. PL spectra with the excitation wavelength at 400 nm and photoluminescence excitation (PLE) spectra with emission wavelength at 570 nm for (c) PhDOT and (d) U:PhDOT. PL spectra of (e) PhDOT and (f) U:PhDOT with different excitation wavelengths (350, 400, 450, and 530 nm).

Both Ph:DOTs and U:PhDOTs displayed the same steady state optical properties (Figure 3); each carbon dot possessed a single emission peak at 570 nm with a full width at half maxima (fwhm) of 85 nm. Also, absorption properties of quantum dots were very similar; the absorption spectrum of each carbon dot had a Gaussian peak at 440 nm, which corresponded to the band-edge transition, a rarely observed peak in absorption spectrum of carbon based quantum dots that could arise because of the formation of a rigid-crystal structure,^35^ and typical carbon dot absorption peaks at lower wavelengths (below 300 nm). On the other hand, the absorption peak of U:PhDOTs at 440 nm was slightly broader than that of PhDOTs in the wavelength range of 470–540 nm, which was an indication of the formation of new energy states due to surface modification. As in emission and absorption spectra, both of the carbon dots possessed almost identical photoluminescence excitation (PLE) spectra; the PLE spectrum of each carbon dot had a Gaussian single peak at 440 nm, which completely resembled the band-edge transition peak in the absorption spectrum of carbon dots. It should be noted that the PLE spectrum of U:PhDOTs did not broaden, as was observed in the absorption spectra of U:PhDOTs compared to those of PhDOTs, which indicated that the broadening of the absorption region was not due to the formation radiative energy transitions but rather due to the formation of nonradiative energy transitions that could arise as a result of the formation of sub-band gap states. The resemblance between the PLE spectrum and the absorption spectrum showed that the luminescence properties of Ph:DOTs and U:PhDOTs were mainly manipulated by the inner structure of the carbon dot and raised because of band-edge electron transitions. To validate this point, the excitation dependence of the emission properties of both carbon dots was investigated. Many types of carbon dots have excitation dependent emission feature, which occur because of the surface characteristics of carbon dots.^36^ This feature can be controlled by manipulating the surface properties of carbon dots.^36^ On the other hand, PhDOTs and U:PhDOTs did not have excitation dependent emission features; carbon dots had a single emission peak at 570 nm upon excitation with different wavelengths such as 350, 400, and 450 nm. The intensity of the emission was at the highest with excitation at 400 nm, decreased dramatically with excitation at 450 nm, and was lost almost completely at an excitation wavelength of 530 nm. Despite both carbon dots having almost identical steady state optical properties, the quantum yields (QY) of each carbon dot were significantly different. The QY of U:PhDOT was calculated to be 51%, where the QY of PhDOT was 40% upon 400 nm excitation. It should be noted that negative effects of sub-band gap states on the quantum yield of U:PhDOTs were not observed because the quantum yields of carbon dots were compared at 400 nm and the radiative transitions were mainly in the 350–450 nm range, whereas the sub-band gap states were observed around 470–540 nm.

**Figure 4:**
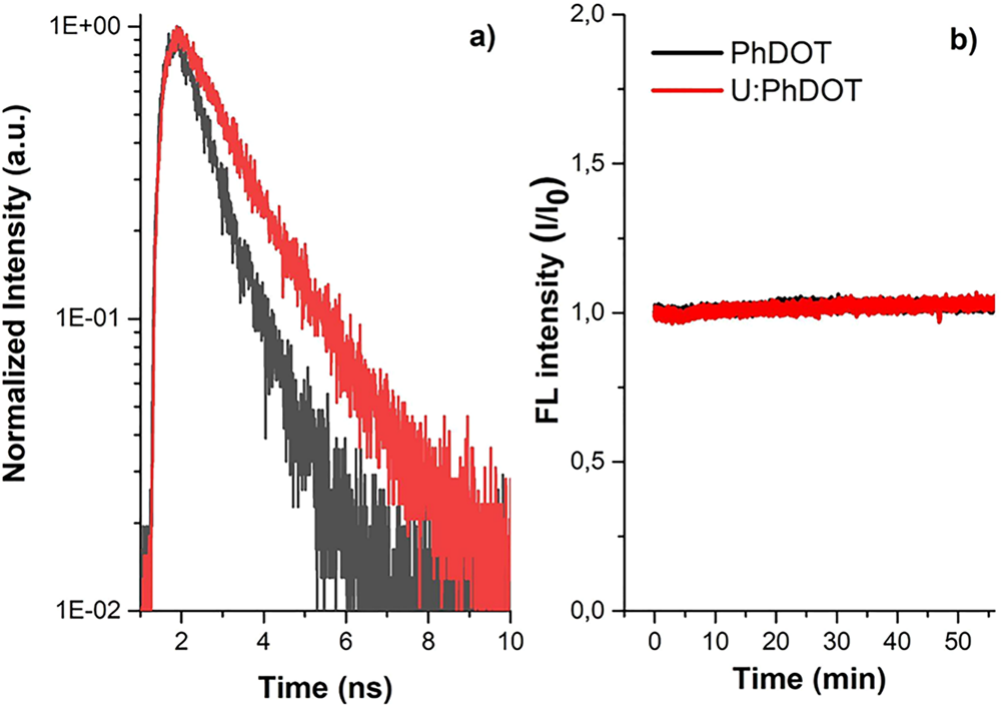
(a) Fluorescence kinetics of CDs at 550 nm upon excitation at 375 nm and (b) photostability of CDs for 1 h upon continuous illumination with λ_exc_ = 350 nm at 25 °C.

To understand the striking difference between the QY of Ph:DOTs and the QY of U:Ph:DOTs, the photostabilities and the fluorescence kinetics of PhDOTs and U:PhDOTs were measured (Figure 4). Both U:PhDOTs and PhDOTs were photostable for at least 1 h under continuous illumination with excitation at 350 nm. To measure the fluorescence kinetics of carbon dots, each carbon dot was excited at 375 nm, and the resulting decay of photons was counted in the 550 ± 15 nm emission range. By measuring the fluorescence kinetics of a fluorescent material, one can calculate the fluorescence lifetime of this material; the fluorescence lifetime of a material reflects the amount of radiative recombination of the excitons, which is observed as fluorescence. A longer fluorescence lifetime means a higher amount of radiative decay, and shorter lifetime means vice versa. PhDOTs had a monoexponential fluorescence decay with an average fluorescence lifetime of 1.1 ns. On the other hand, U:PhDOTs had a considerably longer fluorescence lifetime, around 1.8 ns, compared to PhDOTs. This observation showed that the addition of urea during the synthesis of carbon dots caused surface passivation, which could also be concluded from FTIR and Raman spectra of U:PhDOTs, and the surface passivation of carbon dots led to a decrease in the defect states around the carbon dots that resulted in an increase in the amount of radiative combinations. As a consequence, the QY of U:PhDOTs significantly increased.

**Figure 5:**
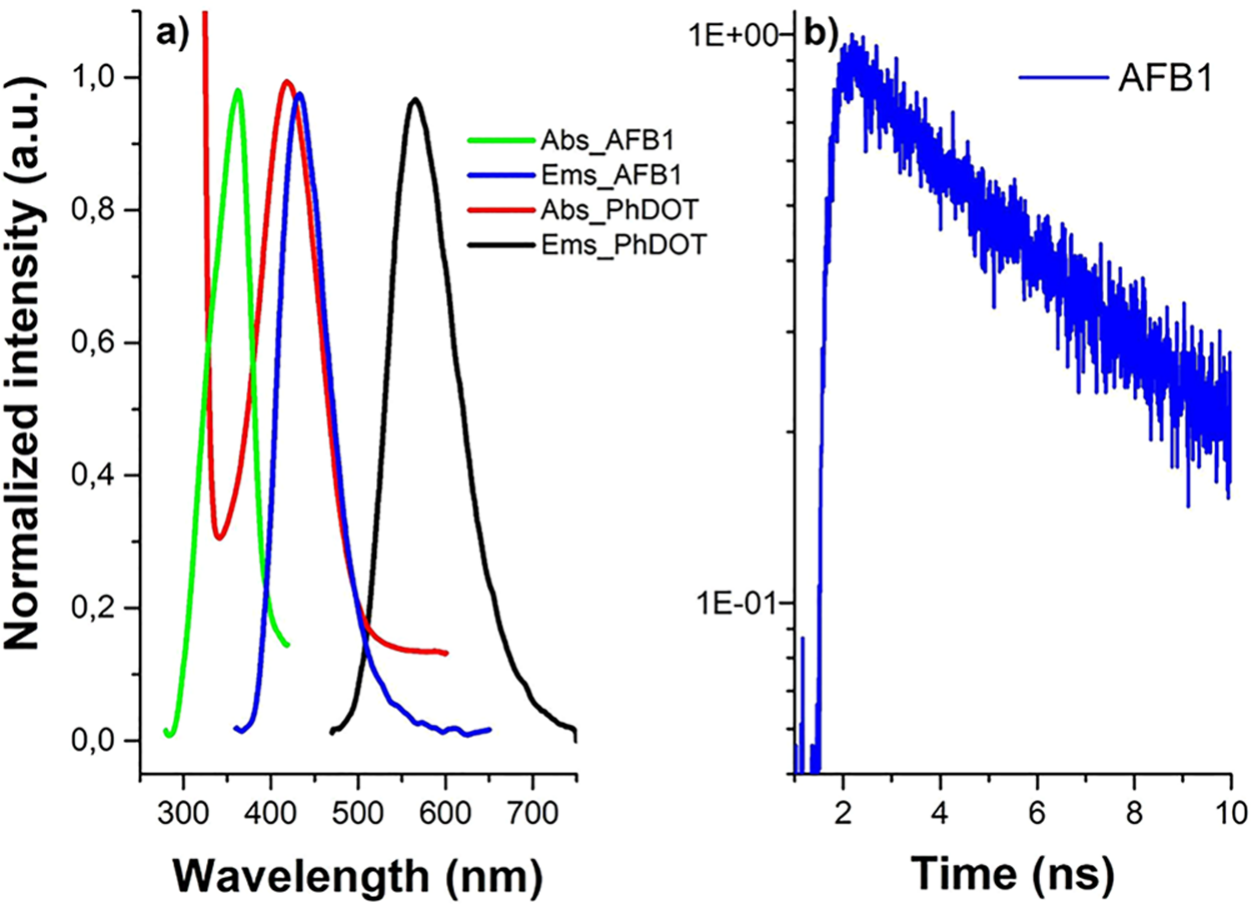
(a) Emission and absorbance spectra of AFB1 and PhDOT. (b) Fluorescence kinetics of AFB1 at 430 nm upon excitation at 375 nm.

Aflatoxin B1 (AFB1) is a biomolecule of interest because of its optical properties. In this study, the interactions between AFB1 and PhDOTs and between AFB1 and U:PhDOTs were studied in detail to understand the potential of using AFB1 as an energy donor to enhance the quantum yield of carbon dots (Figure 5, Table 1). To understand the interaction between carbon dots and AFB1 completely, first steady state and time-resolved optical properties of AFB1 were examined in detail. The absorption spectrum of aflatoxin B1 had a single Gaussian peak at 360 nm. As AFB1 was excited at 350 nm, the emission spectrum of AFB1 displayed a single emission peak at 440 nm. These results showed that the emission peak of AFB1 perfectly overlapped with the band-edge transition peak in the absorption spectra of the carbon dots and the PLE peaks of the carbon dots. The perfect overlap between the emission spectrum of AFB1 and the absorption spectrum of the carbon dots indicated that the optical properties of AFB1 allowed this molecule to be an ideal candidate for a FRET donor for PhDOTs and U:PhDOTs. Also, AFB1 was excited at 375 nm, and the resulting decay of photons was counted in the 430 ± 15 nm emission range. Fluorescence kinetics of AFB1 revealed that the average fluorescence lifetime of AFB1 was 6 ns.

**Table 1:** Steady State Photophsical Properties and Lifetimes of AFB1, PhDOT, and U:PhDOT

	AFB1	PhDOT	U:PhDOT
QY	11%	40%	51%
τ_D_	6 ns	1.1 ns	1.8 ns
Ems λ_max_	440 nm	570 nm	570 nm
Abs λ_max_	360 nm	420 nm	420 nm
fwhm	66 nm	85 nm	85 nm

**Figure 6:**
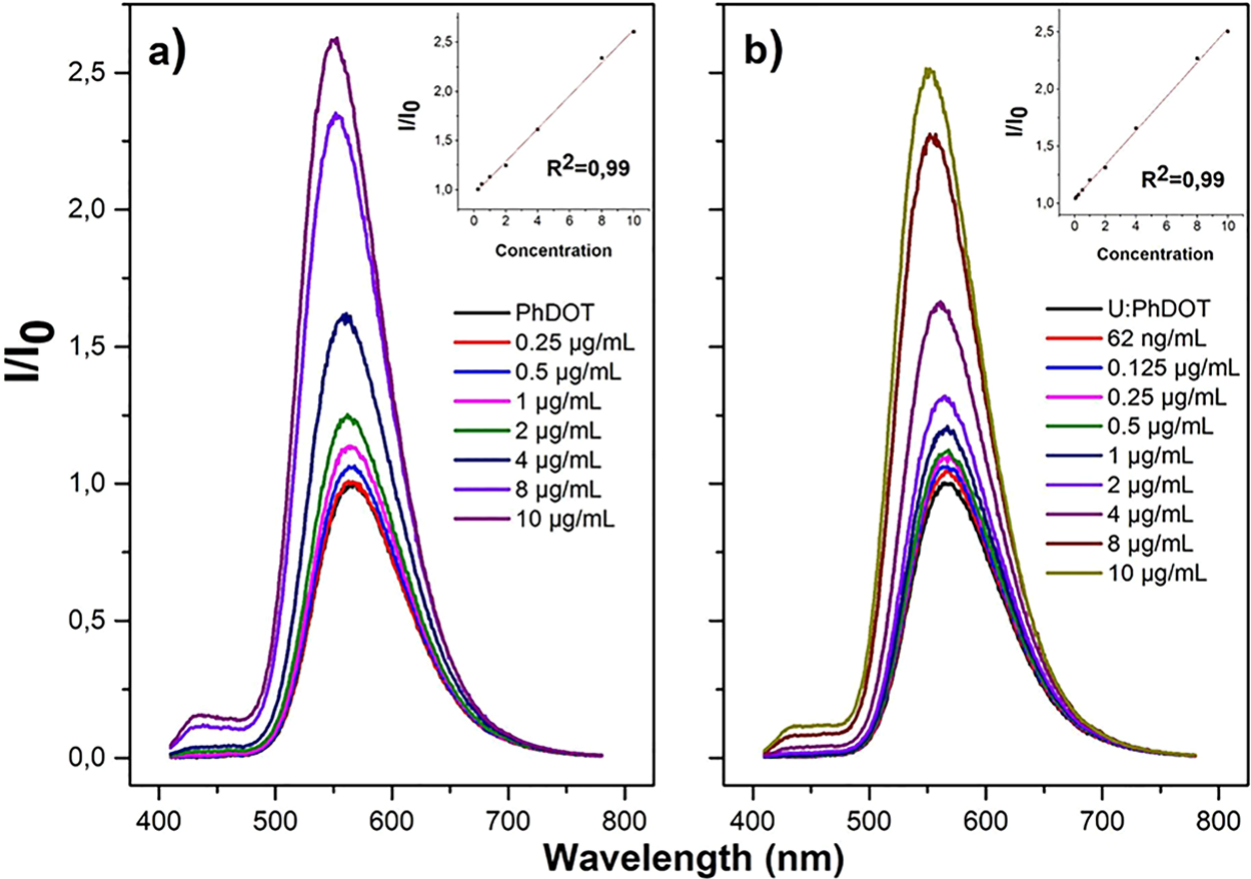
PL spectra of the (a) PhDOT (20 mg/mL)–AFB1 (0.25–10) μg/mL in DI water) interaction (1:3, v/v) and (b) U:PhDOT (20 mg/mL)–AFB1 (62 ng/mL–10 μg/mL in DI water) interaction (1:3, v/v). All samples were excited at 400 nm. Insets of figures correspond to the linear range where *I* is the FL intensity of each component and *I*_0_ is the FL intesities of (a) PhDOT and (b) U:PhDOT.

As AFB1 was added to carbon dot solutions, significant changes in the emission spectrum of each carbon dot type were observed. The emission intensity of each carbon dot type significantly increased with the presence of various concentrations of AFB1 in the carbon dot solution (Figure 6). The increase in the emission intensity of PhDOTs was observed with presence of a minimum 0.25 μg/mL AFB1 concentration. The emission intensity of PhDOT increased linearly (*R*^2^ = 0.99) with the presence of AFB1 with a concentration higher than 0.25 μg/mL and was enhanced by 2.65-fold when the AFB1 concentration was 10 μg/mL. Also, the emission peak of PhDOT slightly shifted toward lower wavelengths, and the emission peak of PhDOTs was observed at 548 nm at a 10 μg/mL AFB1 concentration. A similar case with a few minor but significant differences was observed for the AFB1–U:PhDOT interaction. As was observed in the AFB1–PhDOT interaction, the emission intensity of U:PhDOTs increased linearly (*R*^2^ = 0.99) upon the addition of AFB1 enhanced by 2.45-fold when the AFB1 concentration was 10 μg/mL. However, the increase in the emission intensity was observed with a minimum AFB1 concentration of 62 ng/mL.

**Figure 7:**
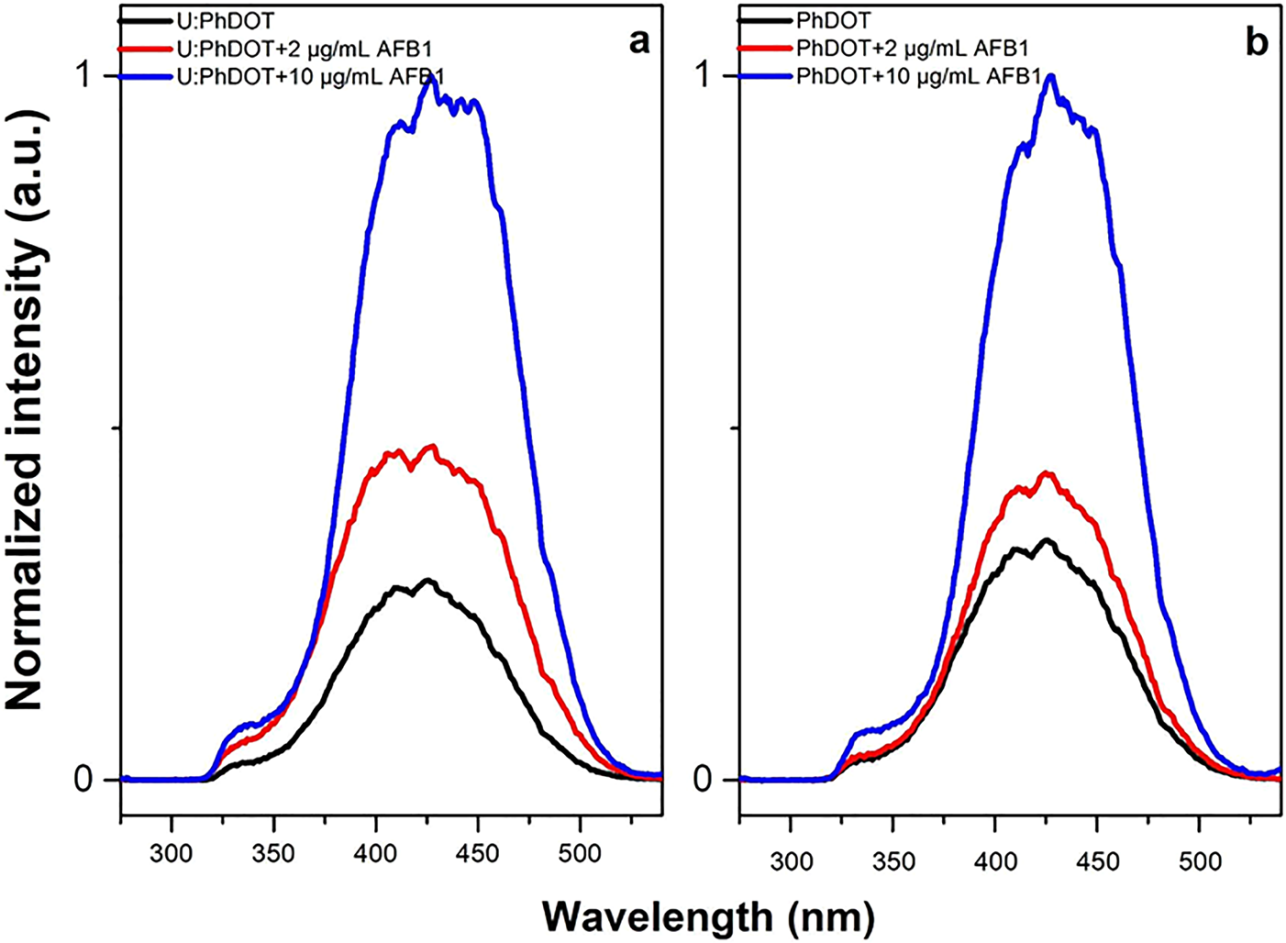
Excitation spectra for (a) U:PhDOT at 570 nm, U:PhDOT + 2 μg/mL AFB1 in water at 566 nm, and U:PhDOT + 10 μg/mL AFB1 in water at 555 nm and (b) PhDOT at 570 nm (maximum emission wavelength), PhDOT + 2 μg/mL AFB1 in water at 564 nm, and PhDOT + 10 μg/mL AFB1 in water at 548 nm.

To understand the reason behind the increase in the fluorescence intensity of yellow emissive carbon dots after interaction with AFB1, steady state PLE spectra and fluorescence kinetics at emission wavelengths of 430 ± 15 nm (AFB1 emission) and 550 ± 15 nm (carbon dots emission) for carbon dot–AFB1 complexes were measured (Figure 7). The intensity of the peak in the PLE spectra of carbon dots gradually increased upon increase in the concentration of AFB1 in the carbon dot solution. These results indicated that the number of radiative transitions increased gradually in the presence of AFB1. However, steady state fluorescence spectroscopy could not provide a more detailed explanation to understand the source of increase in intensity of fluorescence of carbon dots. Two possible mechanisms could trigger the increase: (1) The AFB1 molecules could passivate the surfaces of the carbon dots further and could cause an increase in the quantum yield, as was observed in the U:PhDOT case. (2) The AFB1 molecules acted as an energy donor and increased the quantum yield of the carbon dots. Therefore, to clarify the reason behind the increase in the quantum yield of the carbon dots, fluorescence kinetics of PhDOT–AFB1 and U:PhDOT–AFB1 complexes were measured.

**Figure 8:**
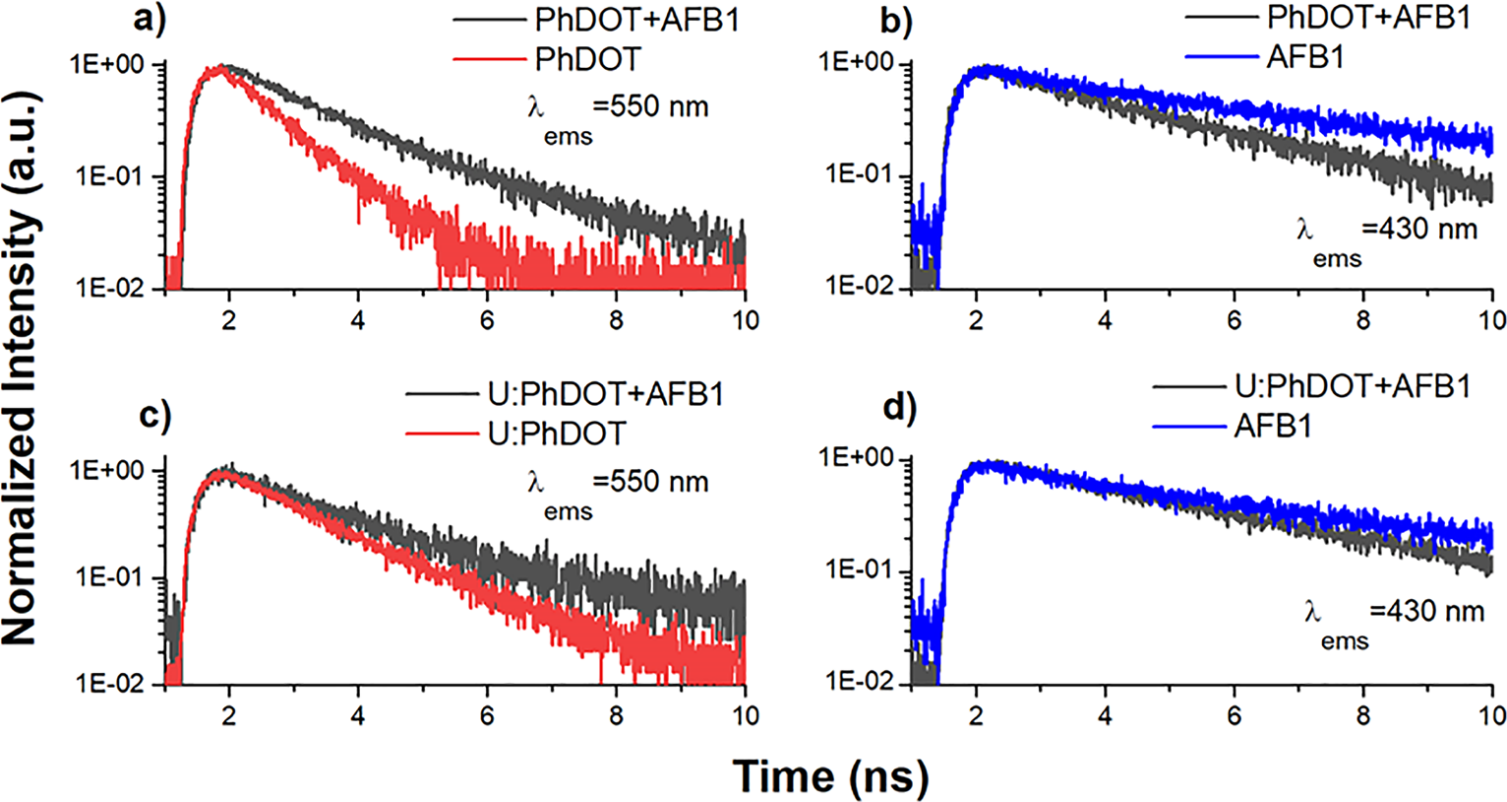
Fluorescence kinetics of (a) PhDOT + AFB1 and PhDOT at 550 nm, (b) PhDOT + AFB1 and AFB1 at 430 nm, (c) U:PhDOT + AFB1 and U:PhDOT at 550 nm, and (d) U:PhDOT + AFB1 and AFB1 at 430 nm. All samples were excited at 375 nm.

The fluorescence decays were collected at two different wavelengths to understand the energy transfer mechanism completely (Figure 8). To understand the change in the fluorescence kinetics of carbon dots, first the fluorescence decays were collected at 550 ± 15 nm, which corresponded to emission of carbon dots. The fluorescence lifetime of carbon dots became significantly longer upon addition of AFB1 (the average fluorescence lifetimes; for PhDOTs before the addition of AFB1:1.1 ns and after the addition of AFB1:2 ns and for U:PhDOTs before the addition of AFB1:1.8 ns and after the addition of AFB1:2.3 ns), which showed that the number of radiative transitions increased upon addition of AFB1 and validated the data obtained from the PLE spectra of carbon dot–AFB1 complexes. On the other hand, the fluorescence lifetime of AFB1, which was measured by measuring the fluorescence decay at an emission wavelength of 430 ± 15 nm, became significantly shorter in carbon dot–AFB1 complexes (the average fluorescence lifetimes for the PhDOT–AFB1 complex of 3.5 ns and U:PhDOT–AFB1 complex of 4 ns; originally for AFB1 it was 6 ns). The main reason for shortening the lifetime of AFB1 in carbon dot–AFB1 complexes was due to the decrease in radiative transitions at 430 ± 15 nm, which were transferred to carbon dots and caused a significant increase in radiative transitions at 550 ± 15 nm. The energy transfer efficiency (*E*) was calculated by using the following formula:
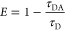
where τ_DA_ is the average fluorescence lifetime of AFB1 in the carbon dot–AFB1 complexes and τ_D_ was the fluorescence of AFB1 alone. *E* for PhDOT–AFB1 was 0.42, whereas *E* for U:PhDOT–AFB1 was 0.33. These results showed that the energy transfer between carbon dots and AFB1 existed regardless of the type of carbon dots; however, the presence of amine groups on carbon dots slightly decreased the efficiency of the energy transfer. On the other hand, it should be noted that the effect of surface passivation on the increase in the quantum yield of carbon dots was much more pronounced compared to the increase in the fluorescence intensity of the carbon dots as a consequence of energy transfer, which could be due to the relatively low quantum yield of AFB1.

## Conclusion

The quantum yield of traditional yellow emissive carbon dots was increased by passivating the surfaces of carbon dots with urea through a simple microwave assisted synthesis method for the first time. Although there was no significant change in the steady state emission properties of carbon dots upon surface passivation, the quantum yield and average fluorescence lifetime of surface passivated carbon dots significantly increased. Then, yellow emissive carbon dots interacted with AFB1, and the emission intensity of carbon dots significantly increased upon this interaction. Fluorescence lifetime measurements revealed that AFB1 acted as an energy donor for carbon dots and caused a significant increase in the fluorescence lifetime and quantum yield of the carbon dots. These results revealed that yellow emissive carbon dots had a remarkable potential to be used as a chemosensor for AFB1 and also AFB1 could be used as a potential emission-intensity booster for yellow emissive carbon dots for biomedical applications such as in vivo cell imaging.

## Supporting Information Available

Hydrodynamic radii of PhDOTs (black) and U:PhDOTs (red) obtained by dynamic light scattering (DLS) (PDF)
