# Supplementary material for: Aflatoxin B1 Acts as an Effective Energy Donor to Enhance Fluorescence of Yellow Emissive Carbon Dots
Source: ACS Omega. 2022 Aug 8;7(33):29297–305. doi: 10.1021/acsomega.2c03498 (PMC9404464; doi:10.1021/acsomega.2c03498)
Supplement: Supplementary file 1 — ao2c03498_si_001.pdf [file ao2c03498_si_001.pdf]

SUPPORTING INFORMATION

Aflatoxin B1 Acts as an Effective Energy Donor to Enhance Fluorescence of Yellow-Emissive-Carbon Dots

Özge Ergüder [a,+], Sultan Şahin Keskin [a,b,c,+], Ilgın Nar [b], Levent Trabzon [c,d] and, Caner Ünlü [a,b,e]*

a. Istanbul Technical University, Department of Nanoscience and Nanoengineering, Maslak, 34469 Istanbul, Turkey

b. Istanbul Technical University Nanotechnology Research and Application Center (ITUNano), Istanbul, Turkey

c. MEMS Research Center, Istanbul Technical University, Istanbul, Turkey

d. Faculty of Mechanical Engineering, Istanbul Technical University, Istanbul, Turkey

e. Istanbul Technical University, Faculty of Science and Letters, Department of Chemistry, 34469, Maslak, Istanbul, Turkey

* Corresponding author.

+ Authors equally contributed to this work.

E-mail address of corresponding author: canerunlu@itu.edu.tr

E-mail address of each author by author order: ozgeerguder@gmail.com, sultan9019@gmail.com, nari@itu.edu.tr, levent.trabzon@gmail.com.

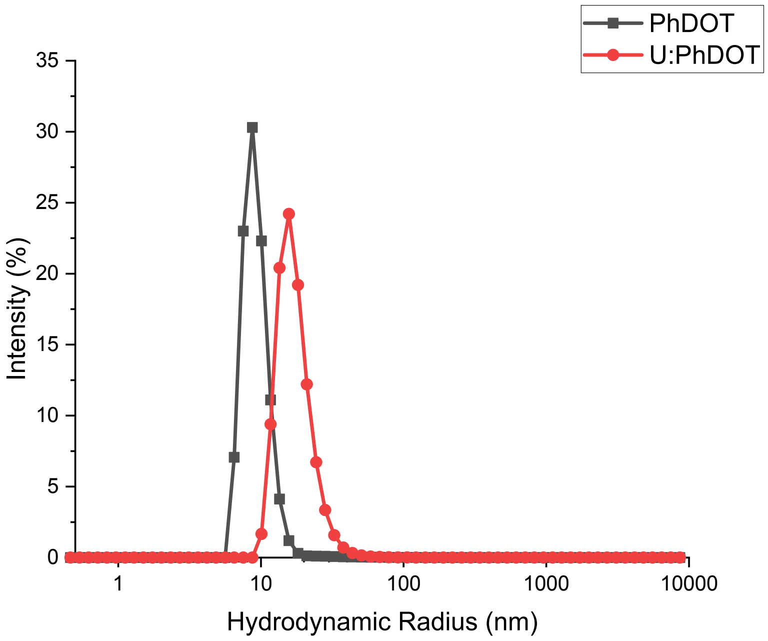

Figure S1. Hydrodynamic radius of PhDOTs (black) and U:PhDOTs (Red)
